# Functional status and its associated factors among community-dwelling older adults in rural Nepal: findings from a cross-sectional study

**DOI:** 10.1186/s12877-021-02286-8

**Published:** 2021-05-25

**Authors:** Saruna Ghimire, Grish Paudel, Sabuj Kanti Mistry, Mahmood Parvez, Binod Rayamajhee, Pravash Paudel, Man Kumar Tamang, Uday Narayan Yadav

**Affiliations:** 1 Department of Sociology and Gerontology and Scripps Gerontology Center, Miami University, 45056 Oxford, OH USA; 2 Centre for Research Policy and Implementation, Biratnagar, Nepal; 3 James P Grant School of Public Health, BRAC University, Dhaka, Bangladesh; 4 Centre for Primary Health Care and Equity, University of New South Wales, Sydney, Australia; 5 School of Optometry, University of New South Wales, Sydney, Australia; 6 School of Population Health, University of New South Wales, Sydney, Australia; 7 Queensland Brain Institute, The University of Queensland, Brisbane, Australia; 8 Torrens University, Sydney, Australia

**Keywords:** Barthel Index, Activities of daily living, Functional Status, Oder adults, Nepal

## Abstract

**Background:**

The high burden of chronic conditions, coupled with various physical, mental, and psychosocial changes that accompany the phenomenon of aging, may limit the functional ability of older adults. This study aims to assess the prevalence of poor functional status and investigate factors associated with poor functional status among community-dwelling older adults in rural communities of eastern Nepal.

**Methods:**

Data on 794 older adults aged ≥ 60 years from a previous community-based cross-sectional study was used. Participants were recruited from rural municipalities of Morang and Sunsari districts of eastern Nepal using multi-stage cluster sampling. Functional status was assessed in terms of participants’ ability to perform activities of daily living using the Barthel Index. Covariates included sociodemographic characteristics, lifestyle factors, and self-reported chronic conditions. A binary logistic regression model was used to investigate factors associated with poor functional status.

**Results:**

The overall prevalence of poor functional status was 8.3 % (male: 7.0 % and female: 9.6 %), with most dependence noted for using stairs (17.3 %), followed by dressing (21.9 %) on Barthel Index. In the adjusted model, oldest age group (odds ratio [OR] = 2.83, 95 %CI: 1.46, 5.50), those unemployed (OR = 2.41, 95 %CI: 1.26, 4.65), having memory/concentration problems (OR = 2.32, 95 %CI: 1.30, 4.13), depressive symptoms (OR = 2.52, 95 %CI: 1.28, 4.95), and hypertension (OR = 1.78, 95 %CI: 1.03, 3.06) had almost or more than two times poor functioning.

**Conclusions:**

One in 12 older adults had poor functional status as indicated by their dependency on the items of the Barthel Index; those in the oldest age bracket were more likely to exhibit poor functional status. We suggest future studies from other geographies of the country to supplement our study from the rural setting for comprehensive identification of the problem, which could guide the development of prevention strategies and comprehensive interventions for addressing the unmet needs of the older adults for improving functional status.

**Supplementary Information:**

The online version contains supplementary material available at 10.1186/s12877-021-02286-8.

## Background

In Nepal, a South Asian country between India and China, the legal provision identifies citizens aged 60 and above as “Senior Citizens” [1]. Nepali older adults’ population is burgeoning; from 3.8 % of the total population in 1950 to 8.6 % in 2019, and is projected to be 10.7 % by 2030 [2]. The population growth rate for older adults (3.5 %) is greater than the overall population growth rate (2 %) of the country [3]. The demographic transition is happening concurrently with the epidemiological transition, resulting in an increased burden of chronic diseases.

The prevalence of chronic health conditions is higher in older age groups [4], in general and also among Nepali older adults [5]. The high burden of prevalent conditions coupled with the various physical, mental, and psychosocial changes that accompany the phenomenon of aging [6], may limit the functional ability of older adults, and consequently, they may need assistance to perform daily activities such as eating, cooking, bathing, moving around, shopping, and managing finances and medications, etc. [7]. These routine activities are used as an indicator of a person’s functional status and are categorized into activities of daily living (ADL) and instrumental activities of daily living (IADL). ADL includes activities related to one’s basic physical needs, such as personal hygiene or grooming, dressing, toileting, transferring or ambulating, and eating, while IADL includes more complex activities of independent living such as managing finances and medications, food preparation, housekeeping, laundry, etc. Notably, disability (dependency on others for basic activities) is commonly defined in terms of difficulty performing ADL and IADL [8]. Globally, over 45 % of older adults aged 60 and over have difficulty performing everyday activities, and over 250 million people experience moderate and severe disabilities [9, 10]. The inability to perform essential everyday activities is also positively correlated with poor quality of life, increased hospitalization [11, 12], increased mistreatment [13], and the need for more physical and social support [14–16]. Inclusion and participation of older adults, with and without disabilities, in the society is in line with the 2030 Agenda for Sustainable Development as well as that of an age-friendly world [10]. Despite this, the prevalence of disability among older people shows an increasing trend [17, 18] and may jeopardize older adults’ full participation in the society.

In Nepal, more than three-fourth of the older adults live in an extended family and receive informal care from family members [2]. Long-term institutional care is almost non-existent in Nepal, and only a handful of facilities are available in urban Nepal. Nevertheless, advocacy for long-term care is rising, especially due to changing family structure and high migration of adult children [19]. Such dynamics are likely to impact older adults’ caregiving seriously, and specifically, those with disabilities are more likely to be affected. Given that the demographic transitions are recent in Nepal, our knowledge of different aspects of aging in Nepal is limited. There is a lack of nationally representative studies on the health and wellbeing of older adults in general, and more specifically, as it relates to disability. Since Nepal is in the early stage of developing policies to address the older population’s health, social and financial needs, understanding their ADL/IADL disability or poor functioning is important for prioritizing areas for policy action. Orienting intrinsic capacity and functional ability-based health system is one of the strategic objectives of Global Strategy and Action Plan on Ageing and Health, 2016 [20]. However, to date, only one study from urban Nepal quantified ADL among Nepali older adults and reported that 8 % of the participants had difficulty with at least one ADL on the Katz scale [21]. Given that more than 80 % of the older adults live in rural Nepal, the previous study [21] is less likely to provide a comprehensive picture.

Our study, conducted among rural older residents, will supplement the previous study and help us to better understand the functional status of Nepali older adults. Various lifestyle-related factors such as age, gender, ethnicity, marital status, educational attainment, occupation, income, alcohol consumption, smoking, different chronic diseases, physical inactivity, depression etc. influence disability among older people [22–26]. Notably, most of these factors could be managed with appropriate clinical and public health intervention. Hence, knowledge of the factors associated with poor functional status may help local stakeholders to identify risk groups and risk factors for targeted interventions. Additionally, to date, there has been no evidence from the southern plain of Nepal, where most of the marginalized communities (Madhesi, Dalits, and Indigenous groups) reside. With this multifold relevance noted, this study aims to investigate the prevalence of poor functional status and its associated factors among community-dwelling older people in rural eastern Nepal.

## Methodology

### Study design and study participants

This study used data on 794 older adults from a previous community-based cross-sectional study conducted in the rural municipalities of two districts of eastern Nepal- Morang and Sunsari. According to the most recent census, which dates back to 2011 [3], with a total of 213,997 households in 1,855 Sq. Km., Morang district had a total population of 965,370, of which 8.2 % were 60 and older. Similarly, in Sunsari districts, there were a total of 162,407 households in a total area of 1,257 sq. Km. and included a total population of 763,487, of which 7.5 % of the population was 60+. Details of the study design are available somewhere else [14, 27]. Briefly, data were collected between January and April in 2018 using a multi-stage cluster sampling technique. First, four rural municipalities from each district were randomly selected, followed by a random selection of five wards in each municipality. From the list of eligible subjects in each municipality, study participants were randomly selected. Inclusion criteria included Nepali nationals age ≥ 60 years and residents of the study area for at least a year. Data were collected by semi-structured face-to-face interviews in Nepali.

### Study variables

#### Dependent variable

Functional status, the outcome variable, is defined in terms of ADL, measured using Barthel Index [28] that assess participants’ independence or dependence on ten everyday activities such as feeding, bathing, dressing, grooming, bowel and bladder control, toilet use, transfers, mobility, and stairs use (Supplemental Table 1). Notably, the index indicates what a patient does and not what a patient could do. The cumulative score for Barthel Index ranges from 0 to 100; a score of > 60 was considered as good functional status (that indicates greater ability to perform everyday tasks without assistance), and ≤ 60 indicates the poor functional status (or greater dependence to perform everyday tasks). The Cronbach alpha for the Barthel Index in the current study was 0.82.

#### Independent variables

For this study, we used the biopsychosocial model of disability, supported by the International Classification of Functioning, Disability, and Health, which integrates both medical and social models of disability. Accordingly, disability and functioning are viewed as outcomes of interactions between health conditions and contextual factors [29]. Consequently, we included sociodemographic characteristics (participants’ age, sex, ethnicity, marital status, educational status, past occupation, and monthly family income in Nepali rupee), lifestyle factors (history of smoking and alcohol use, and physical activity), and health status (depressive symptoms, memory or concentration problem and different chronic conditions) as the explanatory variables. Educational status, assessed in terms of the number of formal schooling, was categorized into with and without any formal schooling/education. History of smoking and alcohol use was assessed as dichotomous (yes/no) responses. Participants were considered to be physically inactive if they reported not engaging in different types of moderate-to-vigorous activities (i.e., walking, jogging, yoga, cycling, exercise, swimming, weightlifting, activities related to the farmhouse, etc.) in the past week; otherwise, they were classified as physically active. Depressive symptoms were assessed using the 15-item Geriatric Depression Scale, details of which are published elsewhere [5]. These independent variables are also described in our previously published paper [30]. Five self-reported chronic conditions, i.e., hypertension, arthritis, cardiovascular diseases, diabetes, and chronic obstructive pulmonary diseases, were assessed by asking participants if a health professional ever told them that they had the condition.

#### Statistical analyses

Frequency (%) distribution of independent variables by functional status is reported, and group differences in frequencies were tested using the Chi-square test. We used binary logistic regression models to explore the factors associated with participants’ poor functional status, where the final model was selected using the backward elimination criteria with the Akaike information criterion (AIC) approach. We used variance inflation factors (VIF) for assessing the multicollinearity of variables. Adjusted odds ratio (OR) and 95 % confidence intervals (95 % CI) of the variables in the final model are reported. All analyses were performed using the Stata software (Version 14.0).

## Results

### Sociodemographic and lifestyle characteristics and health profile of the participants

The mean age of participants was 69.9 years (male: 70.2 ± 8.5; female: 69.7 ± 8.9), and the majority were in their sixties (Table 1). Roughly equal representation of males and females was noted. Participants from Aadiwasi/Janjatis and Madeshi ethnic group represented over 70 % of the participants. The majority of the participants were married (53.5 %), without formal education (80.1 %), unemployed in the past (54.2 %), had a history of smoking (62.2 %), and were physically inactive (77.1 %) (Table 1). About 56 % reported depressive symptoms, and various chronic conditions were prevalent (Table 1).

**Table 1:** Sociodemographic and lifestyle characteristics by functional status (N = 794)

Characteristics		Total (%)	Functional status (%)		
			Good (n = 728, 91.6 %)	Poor (n = 66, 8.3 %)	*P-*value
Age (year)					
	60–69	440 (55.4)	416 (94.6)	24 (5.4)	**< 0.001**
	70–79	235 (29.6)	215 (91.5)	20 (8.5)	
	≥ 80	119 (15.0)	97 (81.5)	22 (18.5)	
Gender					
	Male	400 (50.4)	372 (93.0)	28 (7.0)	0.177
	Female	394 (49.6)	356 (90.4)	38 (9.6)	
Ethnicity					
	Aadiwasi/Janjatis	298 (37.5)	276 (92.6)	22 (7.4)	0.672
	Brahmin/Chettri/Thakur	69 (8.7)	65 (94.2)	4 (5.8)	
	Dalit	157 (19.8)	142 (90.5)	15 (9.6)	
	Madeshi and others	270 (34.0)	245 (90.7)	25 (9.3)	
Marital status					
	Married	425 (53.5)	400 (94.1)	25 (5.9)	**0.008**
	Without partner^a^	369 (46.5)	328 (88.9)	41 (11.1)	
Educational status					
	Without formal education	636 (80.1)	583 (91.7)	53 (8.3)	0.966
	With formal education	158 (19.9)	145 (91.8)	13 (8.2)	
Past occupation					
	Employed	364 (45.8)	351 (96.4)	13 (3.6)	**< 0.001**
	Unemployed	430 (54.2)	377 (87.7)	53 (12.3)	
Family monthly income^b^					
	NRP ≤ 5,000	381 (48.0)	348 (91.3)	33 (8.7)	0.921
	NRP 5,000 - ≤ 10,000	145 (18.3)	134 (92.4)	11 (7.6)	
	NRP > 10,000	268 (33.8)	246 (91.8)	22 (8.2)	
History of smoking					
	No	300 (37.8)	281 (93.7)	19 (6.3)	0.115
	Yes	494 (62.2)	447 (90.5)	47 (9.5)	
History of alcohol drinking					
	No	504 (63.5)	458 (90.9)	46 (9.1)	0.273
	Yes	290 (36.5)	270 (93.1)	20 (6.9)	
Physical activity					
	Inactive	612 (77.1)	557 (91.0)	55 (9.0)	0.207
	Active	182 (22.9)	171 (94.0)	11 (6.0)	
Memory or concentration problem					
	No	486 (61.2)	465 (95.7)	21 (4.3)	**< 0.001**
	Yes	308 (38.8)	263 (85.4)	45 (14.6)	
Depressive symptoms					
	No	351 (44.2)	339 (96.6)	12 (3.4)	**< 0.001**
	Yes	443 (55.8)	389 (87.8)	54 (12.2)	
Hypertension					
	No	541 (68.1)	507 (93.7)	34 (6.3)	**0.002**
	Yes	253 (31.9)	221 (87.4)	32 (12.7)	
Arthritis					
	No	463 (58.3)	435 (94.0)	28 (6.1)	**0.006**
	Yes	331 (41.7)	293 (88.5)	38 (11.5)	
Cardiovascular disease					
	No	775 (97.6)	711 (91.7)	64 (8.3)	0.723
	Yes	19 (2.4)	17 (89.5)	2 (10.5)	
Diabetes					
	No	752 (94.7)	689 (91.6)	63 (8.4)	0.778
	Yes	42 (5.3)	39 (92.9)	3 (7.1)	
Chronic obstructive pulmonary disease					
	No	672 (84.6)	623 (92.7)	49 (7.3)	**0.014**
	Yes	122 (15.4)	105 (86.1)	17 (13.9)	

^a^ Widowed/divorced/separated/unmarried. ^b^NRP: Nepali rupee; 1 NRP ~ 100 US dollar. Significant p-values are bolded

### Functional status

The overall prevalence of poor functional status was 8.3 % (male: 7.0 % and female: 9.6 %) (Table 1). Supplemental Table 1 reports participants’ responses to each item on the Barthel index of functional status. Participants reported most inability to dress (21.9 %), followed by using stairs (17.2 %), mobility (6.6 %), bathing (6.8 %), grooming (5.6 %), and using the toilet (3.6 %) while other activities were < 2 % prevalent (Supplemental Table 1).

### Independent variables associated with poor functional status

The full model included participants’ sociodemographic and lifestyle characteristics (all variables presented in Table 1). The final model, based on the backward selection using the lowest AIC, is presented in Table 2. Hence, the model is adjusted for all the variables in Table 2. The VIF of the variables included in the model was less than 3.0. In the final model, age, past occupation, memory/concentration problem, depressive symptoms, and hypertension were significantly associated with poor functional status (Table 2). The oldest age group (≥ 80 years) had almost three times higher odds of poor functioning (OR = 2.83, 95 %CI: 1.46, 5.50) compared to the youngest age group (60–69 years). Poor functional status was close to or greater than two times among those unemployed (OR = 2.41, 95 %CI: 1.26, 4.65), had memory/concentration problems (OR = 2.32, 95 %CI: 1.30, 4.13), depressive symptoms (OR = 2.52, 95 %CI: 1.28, 4.95), and hypertension (OR = 1.78, 95 %CI: 1.03, 3.06).

**Table 2:** Association of sociodemographic, lifestyle, and health status with poor functional status

Characteristics		Adjusted odds ratio	95 % CI	*P*
Age (year)				
	60–69	*Ref*		
	70–79	1.45	0.77, 2.76	0.250
	≥ 80	**2.83**	**1.46, 5.50**	**0.002**
Past occupation				
	Employed	*Ref*		
	Unemployed	**2.41**	**1.26, 4.65**	**0.008**
Memory/concentration problem				
	No	*Ref*		
	Yes	**2.32**	**1.30, 4.13**	**0.004**
Depressive symptoms				
	No	*Ref*		
	Yes	**2.52**	**1.28, 4.95**	**0.008**
Hypertension				
	No	*Ref*		
	Yes	**1.78**	**1.03, 3.06**	**0.038**

## Discussion

With an aim to assess the prevalence and correlates of functional status among older adults in rural Nepal, the present study found that one in 12 older adults had poor functional status. Older age, being unemployed, memory/concentration problems, depressive symptoms, and hypertension were associated with poor functional status.

The estimated 8.3 % prevalence of functional dependency in our study is similar to a previous study from urban Nepal [31]. Biological senescence that results in various physical, mental, and psychosocial changes [6] coupled with prevalent chronic conditions may limit the functional ability of older adults to perform everyday tasks. In general, physical functioning diminish with age, and the speed of deterioration accelerates among older adults [32, 33]. Relatedly, we also noted that compared to our youngest participants (60–69 years), those in the oldest age group (≥ 80 years) had an increased likelihood of poor functioning. The positive association of ADL dependency with age was also observed in previous studies conducted in various settings [34–37], and specifically, individuals above 70 years face problems with multiple ADL items [35, 38].

Unemployment, which may serve as a proxy for individuals’ low economic status, was associated with more than doubled odds of poor functioning (or increased ADL dependency). Although we did not find any studies that demonstrated the association between past occupational history and ADL performance among older adults, there is a plausibility that older adults with no past employment are very likely to have poor socioeconomic status, which may increase their unmet needs for social, psychological, and physical wellbeing [39]. In line with the common notion that people with lower socioeconomic status have worse health outcomes and a higher risk of premature mortality [40], the cumulative disadvantage theory posits that socioeconomic disadvantages accumulate during the life course to produce differential health outcomes in later life [41]. From these perspectives, past employment may protect against the decline of physical functioning because of cumulative advantages over the life course. Relatedly, previous studies indicate that older adults with past employment history have a better socioeconomic status for fulfilling daily needs and tend to have a positive attitude towards physical activities, which makes older adults performed better on ADL [42, 43]. Likewise, a longitudinal study conducted among Japanese older adults 70 years and older also indicated that engagement in work might contribute to independence in terms of ADL [44].

The noted increased odds of poor functioning among those reporting depressive symptoms are also supported by previous studies that indicate depressive symptoms impair functional capacity to perform services such as shopping, preparing meals, moving within the community, and taking medications on time [45, 46]. Potential mechanisms underlying the relationship between depressive symptoms and development of functional disability includes enhanced decline of physical functioning over time due to prolonged presence of certain somatic depressive symptoms [47], amplified symptom burden and complications of chronic medical conditions [48], negative health behaviors (such as physical inactivity, obesity, and smoking [49], and non-compliance to various treatment regimens [49]; all of which facilitate the onset of functional disability among the older population.

In line with previous studies that concluded that memory or attention deficiencies are significant predictors of ADL dependency among older adults [50, 51], our participants with memory or concentration problem were at higher odds of poor functioning. Severe memory deficits is one of the earliest and most pronounced symptoms of cognitive impairment related to Alzheimer’s disease, which often causes ADL limitations in older adults [52]. An earlier study also suggested that attentional impairments may be driving impairment in ADL among older people [53].

Hypertension was identified as a risk factor for poor functioning among our study participants which is supported by a number of previous studies [54, 55] that reported greater limitations in ADL among hypertensive older adults. Additionally, evidence suggests that antihypertensive medications can prevent or delay subsequent ADL limitations in older adults [56]. However, there remains controversy regarding the optimal treatment of hypertension in the older population, especially the oldest age group [57]. Therefore, other methods of hypertension control (e.g., lifestyle change) can be prioritized to reduce the higher ADL dependence among the older population.

### Strength and limitations of the study

This study has its own strength and limitations. Strengths of this study include a first study to assess the functional status or ADL dependency among older adults in rural eastern Nepal with a high response rate (> 95 %) and the use of trained enumerators for data collection in the community setting. This study is subjected to certain limitations that included: (a) associations were derived from a cross-sectional study, therefore precludes cause-effect relationship and (b) generalizability of findings is limited to rural settings of Morang and Sunsari district. Additionally, data obtained in this study were self-reported, which may be subjected to social desirability and recall bias. The possibility of reverse causality is also possible given that the relationship between functional status and health conditions could be bidirectional.

## Conclusions

In summary, our study found that one in 12 older adults has poor functional status in rural Nepal, with those in the oldest age bracket more likely to exhibit poor functioning. The study finding highlights the necessity to give special attention to relatively older Nepalese adults, who are at higher risk for poor functioning or ADL dependence. Therefore, evidence-based programs for older adults to improve ADL independence and environments for maintaining independence are warranted [58]. Hypertension and depressive symptoms, both amenable to psychosocial interventions [59], were associated with increased odds of dependency. Therefore, culturally tailored interventions aimed at screening and subsequently treating depression and lifestyle changes for hypertension need to be developed to address these conditions and subsequent functional disability in this population. We suggest future studies from other geographies of the country to supplement our study from the rural for comprehensive identification of the problem, which could guide the development of prevention strategies and comprehensive interventions for addressing the unmet needs of the older adults for improving physical functioning.

## Supplementary Information

**Additional file 1: Supplemental Table 1. **Participants response to 10 items on Barthel Index (BI) of activities of daily living.

## Data Availability

The datasets used and/or analyzed during the current study are available from the corresponding author on reasonable request.

## Abbreviations

ADL: Activities of daily living.
OR: Odds ratio.
CI: Confidence nterval.
IADL: Instrumental activities of daily living.
VIF: Variance inflation factors.
