# Supplementary material for: Functional status and its associated factors among community-dwelling older adults in rural Nepal: findings from a cross-sectional study
Source: BMC Geriatr. 2021 May 25;21:335. doi: 10.1186/s12877-021-02286-8 (PMC8152303; doi:10.1186/s12877-021-02286-8)
Supplement: Supplementary file 1 — Additional file 1: Supplemental Table 1. Participants response to 10 items on Barthel Index (BI) of activities of daily living. [file 12877_2021_2286_MOESM1_ESM.docx]

**Supplemental Table 1: Participants response to 10 items on Barthel Index (BI) of activities of daily living**

| **Activities** | **n** | **%** |
| --- | --- | --- |
| **Feeding** |  |  |
| Inability to perform | 9 | 1.1 |
| Assistance is required | 10 | 1.2 |
| Total independence | 775 | 97.6 |
| **Bathing** |  |  |
| Inability to perform | 54 | 6.8 |
| Assistance is required | 740 | 93.2 |
| **Dressing** |  |  |
| Inability to perform | 174 | 21.9 |
| Assistance is required | 38 | 4.7 |
| Total independence | 582 | 73.3 |
| **Grooming** |  |  |
| Inability to perform | 45 | 5.6 |
| Assistance is required | 749 | 94.3 |
| **Bowels** |  |  |
| Inability to perform | 13 | 1.6 |
| Assistance is required | 15 | 1.8 |
| Total independence | 766 | 96.4 |
| **Bladder** |  |  |
| Inability to perform | 14 | 1.7 |
| Assistance is required | 31 | 3.9 |
| Total independence | 749 | 94.3 |
| **Toilet use** |  |  |
| Inability to perform | 29 | 3.6 |
| Assistance is required | 128 | 16.1 |
| Total independence | 637 | 80.2 |
| **Transfers** |  |  |
| Inability to perform | 15 | 1.8 |
| Assistance is required | 91 | 11.4 |
| Total independence | 688 | 86.6 |
| **Mobility** |  |  |
| Inability to perform | 53 | 6.6 |
| Assistance is required | 39 | 4.9 |
| Total independence | 702 | 88.4 |
| **Stairs** |  |  |
| Inability to perform | 137 | 17.2 |
| Assistance is required | 109 | 13.7 |
| Total independence | 548 | 69.0 |
| **Functional status (based on total BI score)** | | |
| Poor functional status (BI score≤60) | 66 | 8.3 |
| Good functional status (BI score>60) | 728 | 91.6 |
